# The rational treatment of fractures: Use the evidence with caution

**DOI:** 10.4103/0019-5413.77125

**Published:** 2011

**Authors:** Anil K Jain

**Affiliations:** *Editor, Indian Journal of Orthopaedics, and Professor, Orthopaedics, University College of Medical Sciences & GTB Hospital, Delhi, India*

Fracture healing is a biological process in continuum from fracture to healed stage. It involves a sequence of dynamic events which ultimately restores the integrity of the bone and its biomechanical properties.^1^ Bone is the only biological tissue which heals by making bone and the rest of the tissues heal by making a different tissue. The process of healing starts immediately after injury and continues thereafter. The events take place at the cellular level and the summation of these changes are seen as healing. The fracture healing is influenced by physical, chemical and environmental factors.^2^

The severity of damage to the physical environment at the fracture site as a result of injury affects the biological potential for healing. The magnitude of injury decides the insult occurring at the fracture site. The disruption of soft tissues and network of vascular channels makes the bone ends devoid of circulation to a variable length. The more is the force of impact and disruption to the tissues the more is the devascularization. The repair process starts with demineralization of devitalised bone ends and subsequent revascularization. The pleuripotent cells get converted into cartilage cells and later osteoblasts to make a new bone on the scaffold of the devitalised bone and thus finally a woven bone is laid down which in due course gets trabeculated. The fracture ends first get temporarily stabilized by organized fracture hematoma, cuff of fibrocartilage followed by woven bone. The woven bone is seen as fracture callus which continues to remodel for many years. This in short is described as secondary (natural) fracture healing which occurs in the majority of fractures. The primary bone healing occurs where there is a rigid internal fixation. This consists of cutting cones traversing through the devitalised bones which progress across the fracture site directly in a similar way to normal bone remodeling.

The process of healing is a very slow process occurring at cellular levels hence the need to provide a temporary stability by plaster cast for a certain length of time which allows the cascade of events of fracture healing to take place unabated. The normal cascade of events is modified by open injuries, surgical intervention and instability at the fracture site. The length of immobility by plaster cast adds the effects of immobility such as stiffness of joints, secondary to fibrosis in the injured muscles and ligaments hence an exercise regimen is indicated till such time normal joint motions are regained. The stability at the fracture site is of paramount importance. The motion at fracture site within a permissible range (micromotion) stimulates the fracture healing potential while beyond a certain level retards the healing process. The rigid immobilization of fracture is considered unphysiological and micromotion occurring secondary to functional activities encourages osteogenesis^3^. The functional treatment was advocated by Sarmiento to minimize the complications of immobility and stimulate fracture healing by micromotion and physiological cyclical axial loading.^3^

The objective of fracture care is to allow the biological process of healing to occur at a normal pace with minimal hindrance and damage to the injured limb. Since some of the fractures are not aligned by closed manipulation they are treated by open reduction and internal fixation by implants. The surgery has its impact on the natural cascade of fracture healing. The fractures ends are exposed surgically, manipulated and aligned anatomically and internally fixed (stabilized) by intramedullary or extramedullary implants. All this require exposure of bone to certain length and disrupts normal soft tissue with results and disruption of already damaged blood supply. The biological efforts needed for fracture healing are going to be more extensive than healing in a closed fracture. The healing process becomes further complicated when internal fixation fails to achieve relative stability at the fracture site or wide exposure leads to lifting of periosteum and consequent damage to its cambium layer or the fracture fragments are stabilized at the fracture site with a gap of 2 mm or more.^2^

The ideal objective of surgical treatment should be to achieve a better outcome than nonoperative treatment. The outcome of surgical treatment are going to be worse if surgery is not executed perfectly as prescribed by the surgical principles of fracture fixation technique. The open reduction and internal fixation (ORIF) in a closed fracture increases the risk of infection and consequent retardation of fracture healing. A good outcome following ORIF of fractures can only be achieved when the fracture is anatomically reduced, stably fixed, and there is no risk of infection, to allow early postoperative mobilization. When an implant is inserted a race starts between fracture healing and implant failure. In a successful osteosynthesis the fracture unites first before implant fails. The implant has to survive till the biological process of healing is over. The concept of minimal access or least invasive skeletal stabilization is being suggested to minimize the surgical insult to the fracture milieu during internal fixation.

The bad results of ORIF are worse than the worst results of closed reduction. Multiple variables influencing sound osteosynthesis by surgical means include the nutritional state of patient (soil), severity of trauma, complexity of fracture configuration, choice of treatment method, ORIF or closed reduction and internal fixation, quality of instrumentations and implants available, quality of operating room and level of surgical training. If everything is good one can choose any method of treatment. However, if anything is wanting than the surgical treatment of fractures gives bad results than those of conservative treatment.

On a review of the literature we encounter variable conclusions on a clinical problems from different studies and thus a controversy is created. The possible reason for the controversy is dissimilar variables considered for sound and successful fracture outcome. The clinical results are comparable and predictable if identical outcomes are reported by various studies. The evidence based guidelines are relevant when the conclusions drawn from a particular setup are implemented in identical clinical settings. This becomes particularly more relevant when conclusions drawn in a developed country are considered in a primary health centre of a developing country disregarding infrastructure, quality of instrumentation and implants and level of surgical training available at that centre, hence the outcomes of fracture management are different. By the time these differences in infrastructure are understood lots of complications are produced and reported. More nonunions and infected nonunions are being reported because various fractures are operated for relative indications in suboptimal theatre conditions with poor quality instrumentations and implants and surgical principles are not adhered to. Hence it is imperative to talk about rational treatment. It is justified to treat a simple closed fracture tibial by interlocked nailing on the pretext of early weight bearing when it is guaranteed that it would not produce any risk of infection. Even if there is a 1% risk of infection (100% for that poor patient) then closed reduction and functional treatment outweigh the surgical treatment. We should read and use the published literature keeping all variables in mind. The rational treatment of fractures is selection of a particular modality of treatment keeping in mind all variables to achieve a predictable and consistent outcome of fracture treatment. Grahm Apley once wrote “the indication for fixation are not immutable; this if the surgical skills or backup facilities (staff, sterility and equipment) are of low order; internal fixation is indicated only when the alternatives are unacceptable (eg. femoral neck fracture). With average skills and facilities, fixation is indicated when alternative methods are possible but are very difficult or unwise (eg. multiple injuries) and with the highest levels of skills and facilities, fixation is reasonable if it saves time, money or bed”.^4^

We at Indian Journal of Orthopaedics attempt to appraise the readership with the best available evidence on controversies of fracture management. We provide guidelines^5^,^6^ to evaluate the methodology of various types of research reported. We must realize that the evidence reported are based on studies having similar best of infrastructure, instrumentations and surgical training. That evidence will give predictable result in identical level hospitals. When that evidence is to be used in peripheral centres with compromised infrastructure a balance needs to be evolved between gain and risk.
